# The Twelve Tissue Remedies

**Published:** 1888-03

**Authors:** 


THE TWELVE TISSUE REMEDIES OF SCHÜSSLER. By Drs. Wm. Bœricke and W. A. Dewey. Pp. 303; price, $2.50. Hahnemann Publishing House, Philadelphia. 1888.

This work gives the therapeutics of the twelve remedies, their therapeutical application, and, lastly, a good repertory of their symptoms. Among these twelve drugs are some of our greatest polychrests, and all of them are very useful remedies when used according to the Law of the Similars. So far, then, we welcome this book, which seeks to improve our knowledge of important remedies; but when we are told to use them according to the theories of Schüssler, or any one else, we demur. There is only one law of cure; man's theories are worth nothing. In Oldenburg, where he lives, Schüssler is laughed at as a crank; he has no standing there and little practice. Therefore we repeat, although we repudiate Schüsslerism, we welcome this book as a very useful addition to our knowledge of some very important medicines, which are, perhaps, too much neglected.

